# Supplementary figures and images for: Analysis of close associations of uropod-associated proteins in human T-cells using the proximity ligation assay
Source: PeerJ. 2013 Oct 22;1:e186. doi: 10.7717/peerj.186 (PMC3807586; doi:10.7717/peerj.186)

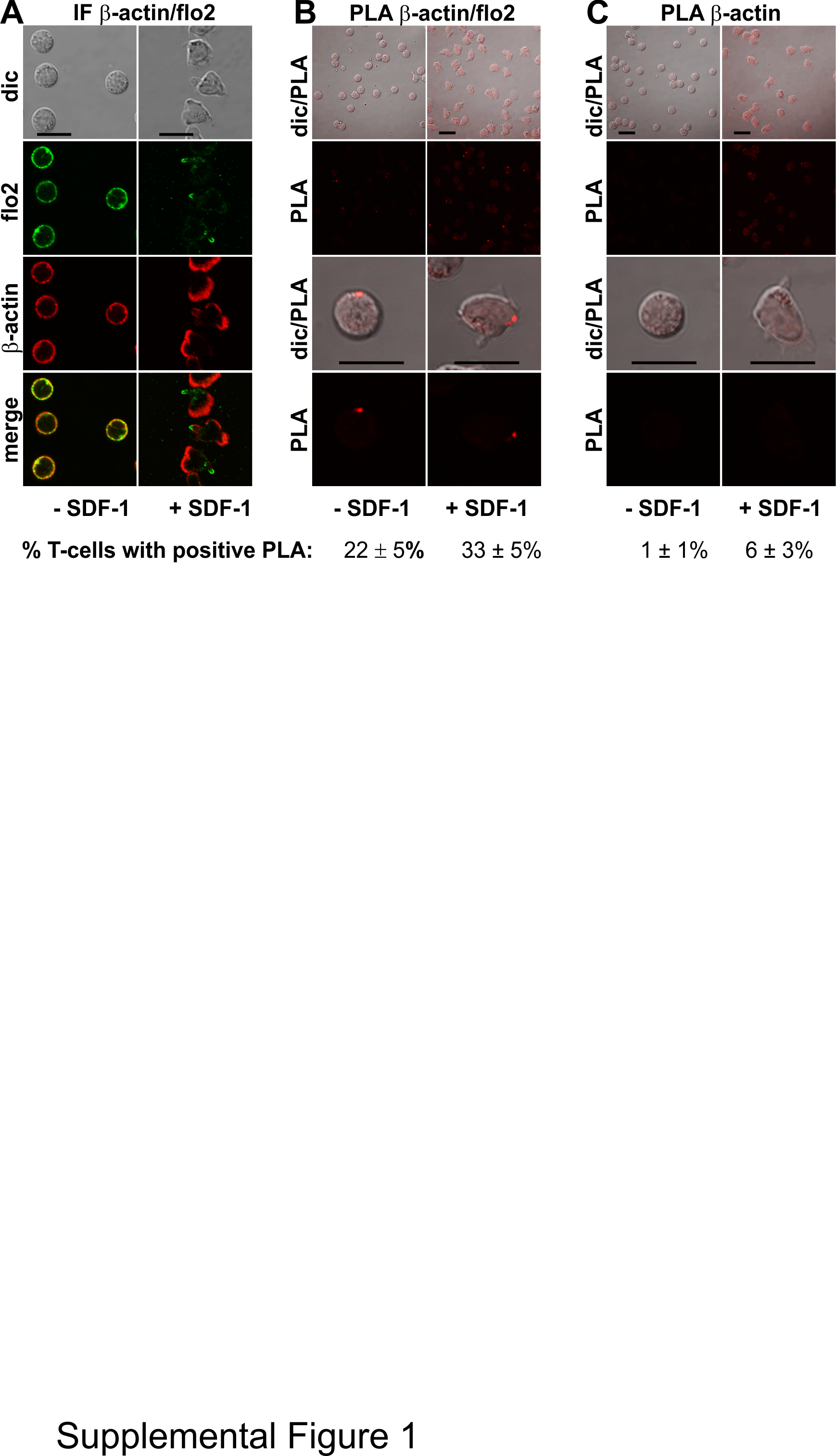

Supplement: Figure S1 — T-cells were preincubated for 30 min at 37°C, followed by a further incubation for 15 min without or with 40 ng/mlSDF-1, fixation with TCA and staining for endogenous β-actin (monoclonal murine antibody) and flotillin-2 (polyclonal rabbit antibody), followed by (A) fluorescently labeled anti-murine and anti-rabbit IgG second antibodies (IF) or (B) PLA probes minus and plus, ligation and amplification. (C) For negative controls, T-cells were treated as described for (B), except that the anti-flotillin-2 antibody was omitted. For (B) and (C),the top panels are overviews at lower magnification whereas in the lower panels single cells are shown at higher magnification. The pictures are representative of 3 experiments. The percentage of cells with one or more red fluorescent dots per cell, indicating positive PLA, was determined for 100 cells per sample and experiment (mean ± sem of 3 experiments). Scale bars, 10 µm. [file peerj-01-186-s001.png]
